# Cervical Cytology and Human Papillomavirus Testing in Adolescent Women: Implications in Management of a Positive HPV Test

**DOI:** 10.1155/2014/165690

**Published:** 2014-03-24

**Authors:** Marilin Rosa, Amir Mohammadi

**Affiliations:** ^1^Department of Pathology and Laboratory Medicine, College of Medicine, University of Florida, Jacksonville, FL 32209, USA; ^2^Anatomic Pathology and Women's Oncology, Moffitt Cancer Center and Research Institute, 12902 Magnolia Drive, Tampa, FL 33612, USA

## Abstract

*Objectives.* Consensus guidelines establish that HPV testing should not be used to manage adolescents with atypical squamous cells of undetermined significance (ASC-US). This study aimed to estimate the impact on follow-up of HPV testing after the first-time ASC-US diagnosis. *Methods.* From January 2009 to December 2010, all ASC-US diagnoses in adolescents were retrieved. *Results.* 1950 cervical cytologies were received from this population and 335 cases (17.1%) were reported as ASC-US. A total of 287 cases were included in the study. Cases were divided into control group (no HPV test; 46 cases) and case group (HPV test performed; 241 cases). On follow-up, in the control group, 43.4% patients had cytology, and 56.6% patients had no follow-up. The case group was divided into negative HPV (60 cases) and positive HPV (181 cases). In the negative-HPV group, 41.7% had cytology and 58.3% had no follow-up. In the positive-HPV group, 41% had cytology, 22% underwent colposcopy, and 37% had no follow-up. Patients with positive-HPV results were more likely to have follow-up than patients in the control and negative-HPV groups (63% versus 43.4% versus 41.7%, resp.). *Conclusions.* HPV infections are common in adolescents. A positive HPV test cannot predict which women will develop carcinoma. Adherence to current guidelines is recommended in this population.

## 1. Introduction

Human papillomavirus is one of the most common sexually transmitted infections in young women with prevalence rates as high as 82% in some populations [1]. The majority of HPV infections are cleared by the immune system in a period of 2 to 3 years [1]. Only persistent infections with oncogenic (high-risk) types have the potential to progress to invasive carcinoma [2].

Cervical cancer is extremely rare in adolescents with only approximately 0.2% of cases of cervical cancer diagnosed under the age of 20 [2]. Therefore, cervical cytology screening and HPV testing are not recommended in this population. Albeit screening guidelines published in 2006 established that HPV test should not be used in women under 21 years of age [3]; in our setting routine cervical cytology screening and HPV testing are commonly performed in this population. The aim of this study was to evaluate the frequency of testing and how a positive HPV test result impacted initial clinical management of patients with first time ASC-US diagnosis.

## 2. Methods

The study was approved by the University of Florida (UF) Institutional Review Board (IRB). Our laboratory receives cervical cytology from community based primary care clinics run by family practice specialized physicians as well as UF-based gynecologists. All gynecologic cytology is processed using ThinPrep (Hologic, Marlborough, MA) liquid based preparation and HPV test is performed on site using the Digene Hybrid Capture II (HCII) method on residual cytologic material.

From January 2009 to December 2010, all cases diagnosed as ASC-US in adolescent patients were retrieved. The inclusion criterion was patients under 20 years of age and first time ASC-US diagnosis. Follow-up data of these cases were collected until 1 year after the initial diagnosis of ASC-US. The one-year period of follow-up was chosen because, according to 2006 screening guidelines (in effect in 2009-2010), adolescents with ASC-US should have repeat cytology at 1 year, and additional tests, including HPV testing, were not expected before that time [3]. The purpose was to prove that those adolescents with HPV test were more prone to additional testing beyond what is recommended for this population. Patients with known previously diagnosed cervical abnormalities were excluded from the study since clinical history of dysplasia may influence clinician's decision. HPV test was performed upon physician's request (test requested as reflex if ASC-US).

Enrolled patients were divided into two groups: (1) control group (HPV test was not performed/requested); (2) HPV group (HPV test was performed). This latter group was further divided into positive-HPV group and negative-HPV group. Follow-up within 1 year was classified into three possibilities as follows: (1) repeat cytology, (2) colposcopy with biopsy, and (3) no follow-up.

## 3. Results

During the study period, 1950 Papanicolaou tests (Pap test) were received from this patient population. Three hundred thirty-five cases (17%) were reported as ASC-US. Two hundred eighty-seven cases (287) met the inclusion criteria, meaning that the patient did not have history of previous abnormal cytology in our electronic medical record system and no history of dysplasia was provided on the pathology requisition. One hundred fifty-nine (55%) of the patients had at least one follow-up within one year. One hundred fourteen (71%) of these patients belong to the positive-HPV group. Seventy-five percent (75%) of patients with ASC-US in which the high-risk HPV test was performed had a positive result (181/241 cases).

### 3.1. Control Group

The control group included 46 cases with ASC-US. As a follow-up, in the control group, 20 patients had repeated cytology (43.4%), 26 patients had no follow-up (56.6%), and no patient underwent colposcopy. One patient was diagnosed with high grade squamous intraepithelial lesion (HSIL) on follow-up cervical cytology. This patient did not undergo colposcopy during the study period. A subsequent Pap test performed one year later revealed ASC-US. HPV test was negative.

### 3.2. HPV Group

The positive-HPV and negative-HPV groups included 181 and 60 patients, respectively.

In the positive-HPV group, a total of 114 (63%) patients had follow-up. Seventy-four patients had repeated cytology (41%), 40 patients underwent colposcopy (22%), and 67 had no follow-up (37%). Four patients were diagnosed with cervical intraepithelial neoplasia 2 (CIN-2) on follow-up biopsy during colposcopy. Of these patients, one underwent cone biopsy (conization) with the diagnosis of cervical intraepithelial neoplasia 3 (CIN-3), one patient underwent loop electrosurgical excision procedure (LEEP) with the diagnosis of CIN-2, one patient did not received surgical treatment and had a subsequent negative Pap test, and one patient was lost to follow-up.

In the negative-HPV group, 25 patients had cytology follow-up (41.7%), 35 patients had no follow-up (58.3%), and no patient underwent colposcopy. No cases of HSIL were found in this group.

Invasive carcinoma was not seen in our study during the follow-up period.

Data are summarized on Tables 1 and 2.

## 4. Discussion

Risky behavior is a major cause of morbidity and mortality among adolescents [1, 3]. According to the CDC's “Youth Risk Behavior Surveillance—United States, 2011,” nationwide, 6.2% of students had had sexual intercourse for the first time before the age of 13 and 15.3% of high school students had had sexual intercourse with four or more persons during their life [4]. In addition, the presence of more active squamous metaplasia and a more exposed transformation zone towards the ectocervix in adolescents are factors that favor HPV infection and replication [1].

Screening guidelines published in 2006 established that adolescents with ASC-US should be followed up with annual cytology. In addition, HPV testing should not be used under the age of 21 years and if it is inadvertently performed, a positive result should not influence management [3, 5]. Furthermore, it is recommended that adolescents with a histological diagnosis of CIN 1, CIN 2, and CIN 2,3 (meaning cases where the pathologist equivocated between CIN 2 and CIN 3) may be followed with observation rather than invasive interventions. For biopsy confirmed CIN 3, treatment is indicated [5].

In 2012, updated guidelines for screening and management of cervical dysplasia were published. These recent guidelines recommend no screening in adolescents. Additionally, since cervical cancer risk remains low through age 25 years, HPV infections are common, and lesions often regress; a less intensive management for young women with abnormal cytology was advised [6].

Some of the reasons behind these recommendations included the current knowledge that HPV infection, ASC-US, and even LSIL are common in adolescents. There is currently good evidence that in adolescents more than 90% of HPV infections, LSIL cytology, and CIN 1 lesions regress within 3 years [5]. Therefore, observation over intervention for adolescents with LSIL and CIN 1 is strongly recommended. Additionally, more than 70% of adolescents with ASC-US are HPV positive (75% in our study); thus HPV testing is not useful for triage in adolescent populations. Many adolescents experience multiple sequential HPV infections, and a repetitively positive HPV test may represent repeated transient infections rather than a persistent infection [5, 7–15]. Certainly, in the era of HPV vaccination, guidelines and role of testing will significantly change.

After 2006 guidelines publication and dissemination, we expected the use of HPV test in adolescents with ASC-US to be totally discontinued or at least gradually decreased. However, in our study, the majority of ASC-US girls (83.9%) had HPV test performed after first known ASC-US diagnosis. Patients with positive-HPV results were more likely to have follow-up in comparison with the control group and the negative-HPV group (63% versus 43.4% versus 41.7%, resp.). Only patients with a positive-HPV result underwent colposcopy.

**Figure 1:**
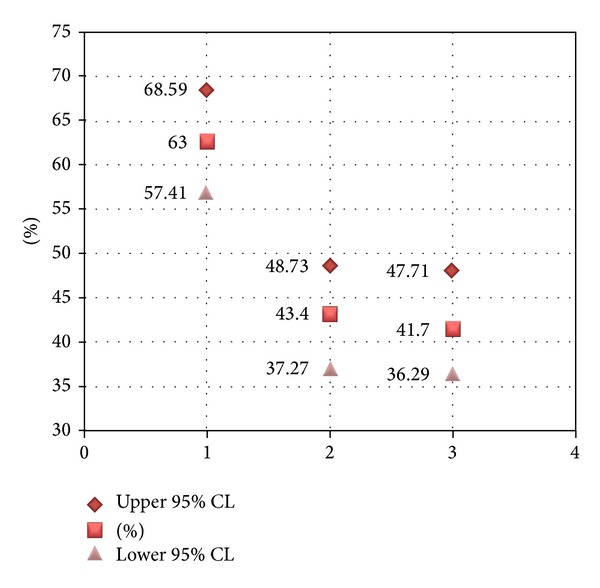
Comparison of HPV-positive group (1), control group (2), and HPV-negative group (3) using 95% confidential intervals (CL) indicates a significant difference in follow-up between the HPV-positive group and the other two groups, while no difference was detected between the control and the HPV-negative groups.

Based on the results from the case group (HPV tested patients) on follow-up, the positive predictive value (PPV) of a positive-HPV test for significant dysplasia (moderate or severe dysplasia) in our population was only 3.5% and 0% for invasive carcinoma. For statistical analysis, the three groups were compared using 95% confidence intervals calculated using SPSS 17.0 (SPSS. Chicago, IL). Results showed that there was a significant difference in follow-up between the HPV-positive group and the other two groups. No difference was detected between the control and the HPV-negative groups (Figure 1). This may indicate that clinicians, at least in our setting, strongly rely on HPV results for clinical decisions in this population.

Although five patients were detected with HSIL, CIN 2, and CIN 3 during the study period (Table 2), there is no data to support that screening under the age of 21 years prevents invasive cervical cancer [3, 5]. These patients probably had previous abnormal cytology not recorded in our system or were infected with high-risk HPV virus long before being enrolled in the study. On follow-up, two of these patients had subsequent negative results. These findings might support the recent notion that even high-grade lesions (at this age) can be carefully observed since they may regress if given the opportunity. Also, although these patients should be carefully observed, other modalities and not HPV testing should be used since HPV test results do not provide significant information about the real risk for developing or having CIN 3 or carcinoma. Given that HPV test has very low PPV in adolescents, annual cytology would suffice for the detection of these rare high-grade lesions before they become invasive. Taken into consideration that the cost of HPV testing is near 300 dollars (280 dollars at our institution), unnecessary testing will also increase healthcare cost.

In spite of the discussion, it is valid to mention here that although HPV testing and cervical screening are currently not recommended in adolescents, guidelines are developed to assist clinicians in patient management; however they may not apply to all clinical scenarios [6], meaning that clinical history such as early onset of sexual activity and previous dysplasia is important in screening and follow-up determinations.

Due to the results of the study and after the 2012 guidelines were published, our department joined efforts with the Department of Obstetrics and Gynecology (OB-GYN) and started an educational campaign targeted to educate community physicians about the adequate use of cytology and HPV and promote the use of current guidelines while treating adolescents. Additionally, our cytology requisition form was changed to modify the HPV options and accommodate current accepted guidelines. A prospective study is currently undergoing to evaluate the impact of 2012 guidelines and our efforts to disseminate them (data not available).

## 5. Conclusions

Since low- and high-risk HPV infections are highly prevalent in adolescents and are commonly transitory, HPV testing is of little predictive value and should not be used to decide the management of adolescent with ASC-US cytology. An abnormal result cannot determine which patients currently may have or will develop cancer in the future and can result in more harm than benefit for the patient. Overuse of diagnostic and therapeutic procedures at this young age may increase morbidity and compromise reproduction in the future due to cervical incontinence. Our experience highlights a significant health care problem that is probably not limited to our setting. Education and adherence to guidelines will help reduce health care costs and emotional and physical patient harm without compromising early cervical cancer detection and treatment.

## Figures and Tables

**Table 1:** Follow-up data by group.

Follow-up	Control group	Positive HPV	Negative HPV
Cervical cytology	20 (43.4%)	74 (41%)	25 (41.7%)
Colposcopy with biopsy	0	40 (22%)	0
No follow-up	26 (56.6%)	67 (37%)	35 (58.3%)

Total	20/46	114/181	25/60

**Table 2:** Follow-up diagnoses by group.

Follow-up diagnosis	Control group	Negative-HPV	Positive-HPV
Negative	11	19	41
ASC-US	3	4	26
LSIL/CIN-1	5	2	43
HSIL/CIN-2/3	1	0	4

Total	20 patients	25 patients	114 patients

ASC-US: atypical cells of undetermined significance; LSIL: low grade squamous intraepithelial lesion; CIN: cervical intraepithelial neoplasia; HSIL: high-grade squamous intraepithelial lesion.
